# Special Issue: Intramolecular Hydrogen Bonding 2017

**DOI:** 10.3390/molecules22091521

**Published:** 2017-09-11

**Authors:** Steve Scheiner

**Affiliations:** Department of Chemistry and Biochemistry, Utah State University, Logan, UT 84322-0300, USA; steve.scheiner@usu.edu; Tel.: +1-435-797-7419

Even after more than a century of study [1,2,3,4,5,6], scrutiny, and detailed examination, the H-bond continues [7,8,9,10,11,12] to evoke a level of fascination that surpasses many other phenomena. Perhaps it is the ability of the simple H atom, with but a single electron, to act as a glue that maintains contact between much more complicated species. Or it might be its geometry, which prefers to hold the bridging proton on a direct line between the two heavy atoms. Not to be ignored are the spectral features of the H-bond: the large red shift of the stretching frequency of the covalent A–H bond, coupled with its intensification, or the downfield shift of the proton’s NMR signal. Yet study of this bond is far from complete, with one surprise after another continuing to emerge. As it turns out, the aforementioned red shift, for example, long considered as the trademark of this bond, is not so characteristic after all. H-bonds that shift in the opposite direction, to the blue, have been observed [13,14,15,16] in a variety of systems. The long held belief that only very electronegative atoms like F, O, and N can participate in these bonds has been shattered, as one atom after another, S and Cl and even metals to name just a few, have been added [17,18,19,20] to the rapidly growing list.

While H-bonds are typically conceived as interactions between pairs of molecules, there is no reason whatsoever why they cannot occur within the confines of a single molecule. Indeed, such intramolecular H-bonds are treated as fundamental underpinnings of the structure and function of such stalwart biomolecules [21,22,23] as proteins, nucleic acids, and carbohydrates. Despite their wide occurrence, intramolecular H-bonds have been studied far less than their intermolecular cousins. One reason for this less robust literature is a set of complications that cloud the interpretation of the data. In the avenue of quantum calculations, for example, a bulwark of the characterization of any interaction such as an H-bond is the energy required to break it. However, the definition of the H-bond energy for an intramolecular H-bond is fraught with complications, and has engendered a number of different approaches, one could almost say ‘tricks’, to seek an accurate and reliable means of its estimation.

It is to this subject of intramolecular H-bonds that this issue is devoted. There are a number of review articles that summarize the current state of the art in various disciplines. Oksanen et al. explain how neutron crystallography can be applied to such bonds in the context of large macromolecules [24]. Hansen and Spanget-Larsen review progress in the application of NMR and IR in the context of particularly strong H-bonds [25]. The manner in which quantum calculations can mesh with experimental approaches is summarized by Siskos et al. [26]. The involvement of one particular element, F, is described in some detail by Mishra and Suryaprakash [27], and Sobczyk et al. review a select group of intramolecular H-bonds [28].

Aside from these review articles, the issue contains a wide variety of reports of new studies. Quantum calculations are an important means to analyze interactions such as these and the influence of such calculations represents a common theme in much of the work described here. Quiquempoix et al. look at levoglucosan and its derivatives [29], while variously substituted aminobenzamides come under the scrutiny of Mphahlele et al. [30]. Mammino considers the relevance of these interactions to an antioxidant [31]; the possibility of multiple intramolecular H-bonds, and their relevance to regioisomers, is discussed by Martínez-Cifuentes et al. [32]. Rusinska-Roszak [33] focuses on the relation between these bonds and aromaticity, while lactams are the subject of the contribution by Mejia et al. [34]. Bilonda and Mammino apply their calculations to quinine [35], and the naphthalene series is examined by Sanchez-Sanz et al. who compare H-bonds to their chalcogen analogues [36]. Scheiner explains how quantum calculations of NMR data can provide unambiguous evidence of H-bonds, as well as their strength [37]. In addition to quantum calculations, methods of dynamics can also be useful, as explained by Huang et al. [38]. 

It is hoped that this select list of reports and review articles will provide the reader with some perspective on the field of intramolecular H-bonds. Some of this work may perhaps inspire others to pursue threads that remain open in this area, with many lingering questions that beg for answers.
